# Corticosteroids in Pediatric Heart Surgery: Myth or Reality

**DOI:** 10.3389/fped.2018.00112

**Published:** 2018-04-20

**Authors:** Daniel P. Fudulu, Ben Gibbison, Thomas Upton, Serban C. Stoica, Massimo Caputo, Stafford Lightman, Gianni D. Angelini

**Affiliations:** ^1^Department of Cardiac Surgery, Bristol Heart Institute, Bristol, United Kingdom; ^2^Henry Welcome Laboratories for Integrative Neuroscience and Metabolism, School of Clinical Sciences, University of Bristol, Bristol, United Kingdom; ^3^Cardiac Anesthesia and Intensive Care, Bristol Heart Institute - University Hospitals Bristol NHS Foundation Trust, University of Bristol, Bristol, United Kingdom; ^4^Department of Congenital Cardiac Surgery, Bristol Royal Hospital for Children - University Hospitals Bristol NHS Foundation Trust, University of Bristol, Bristol, United Kingdom

**Keywords:** corticosteroids, clinical outcomes, relative adrenal insufficiency, deep hypothermic circulatory arrest, pediatric heart surgery, cardiopulmonary by-pass

## Abstract

**Background:** Corticosteroids have been administered prophylactically for more than 60 years in pediatric heart surgery, however, their use remains a matter of debate. There are three main indications for corticosteroid use in pediatric heart surgery with the use of cardiopulmonary bypass (CPB): (1) to blunt the systemic inflammatory response (SIRS) induced by the extracorporeal circuit; (2) to provide perioperative supplementation for presumed relative adrenal insufficiency; (3) for the presumed neuroprotective effect during deep hypothermic circulatory arrest operations. This review discusses the current evidence behind the use of corticosteroids in these three overlapping areas.

**Materials and Methods:** We conducted a structured research of the literature using PubMed and MEDLINE databases to November 2017 and additional articles were identified by cross-referencing.

**Results:** The evidence suggests that there is no correlation between the effect of corticosteroids on inflammation and their effect on clinical outcome. Due to the limitations of the available evidence, it remains unclear if corticosteroids have an impact on early post-operative outcomes or if there are any long-term effects. There is a limited understanding of the hypothalamic-pituitary-adrenal axis function during cardiac surgery in children. The neuroprotective effect of corticosteroids during deep hypothermic circulatory arrest surgery is controversial.

**Conclusions:** The utility of steroid administration for pediatric heart surgery with the use of CPB remains a matter of debate. The effect on early and late outcomes requires clarification with a large multicenter randomized trial. More research into the understanding of the adrenal response to surgery in children and the effect of corticosteroids on brain injury is warranted.

## Introduction

The introduction of the cardiopulmonary bypass (CPB) circuit in the mid-50s made the surgical treatment of intracardiac lesions possible and led to a rapid progress in the field of cardiac surgery [1]. However, CPB is also known to provoke a systemic inflammatory response (SIRS) due to the contact of blood with the extracorporeal circuit, ischemic reperfusion injury of the heart or endotoxemia due to increased gut permeability. This systemic activation is potentially beneficial because it triggers an immune response that could prevent infection and promote healing, but can also prove detrimental and thus result in organ dysfunction and even death [2]. Therefore, since the introduction of the CPB, various strategies have been employed to modulate this SIRS with an aim to improve clinical outcomes. Such strategies include the use of glucocorticoids, aprotinin, antioxidants, and miniaturized or heparin-coated bypass circuits [3]. Furthermore, in pediatric heart surgery, the modulation of SIRS is of greater importance because it is believed that the inflammatory response is augmented by the surface of the extracorporeal circuit relative to the reduced circulating blood volume, the more frequent use of the deep hypothermic circulatory arrest (DHCA) and the more pronounced hemodilution [4] compared with procedures in adults. The use of corticosteroids in cardiac surgery began in the 1960s [5] and according to several current surveys of clinical practice, corticosteroids are still widely used in pediatric heart surgery that involves CPB [6, 7]. By contrast, in adult heart surgery, the use of prophylactic corticosteroids is no longer routine because of no clear evidence backing their administration. The DECS trial recruited 4,494 adult patients undergoing CPB surgery and found no impact of a single intraoperative dose of dexamethasone (1 mg/kg) on the composite end-point of death, myocardial infarction, stroke, renal failure and respiratory failure at 30 days. However, in the same study, dexamethasone was associated with reductions in postoperative infection, duration of mechanical ventilation and length of intensive care and hospital stays [8]. In the largest study of corticosteroids vs. placebo in adults to date, the SIRS trial, 7,507 patients were randomly assigned to methylprednisolone 250 mg at anesthetic induction and 250 mg at the initiation of CPB, or placebo [9]. Corticosteroids had no impact on the risk of death or major morbidity including infection, length of hospital, intensive care stay, respiratory, or renal failure.

The prophylactic use of corticosteroids in pediatric cardiac surgery population continues to be a matter of debate likely due to the lack of well-designed, large randomized controlled trials (RCTs) that can detect a treatment effect in the context of the current low perioperative mortality and morbidity. However, corticosteroids are also given in pediatric heart surgery to protect against the so-called *relative adrenal insufficiency* that can accompany the acute stress of surgery [10, 11]. Due to a lack of basic understanding of hypothalamic-pituitary-adrenal axis physiology during and after pediatric heart surgery, the evidence is limited in this area [12]. Finally, another potential use of corticosteroids in pediatric heart surgery is for their potential neuroprotective effect during DHCA procedures. In the current review, we will discuss the evidence and controversies around these three main indications of steroid use in pediatric heart surgery. We will discuss these topics separately, although their pathogenesis is interconnected.

## Materials and methods

We conducted a structured research of literature using PubMed and MEDLINE databases. The search strategy included a combination of the terms: “steroid,” “glucocorticoid,” “corticosteroid,” “dexamethasone,” “hydrocortisone,” “methylprednisolone,” “pediatric,” “pediatric,” “heart surgery,” “cardiac surgery,” “children,” “neonates,” “deep hypothermic circulatory arrest,” “adrenal.” The last search was conducted in November 2017. Additional articles were identified by cross-referencing from author reference lists and published review papers. We have included all articles assessing the effect of corticosteroids on inflammation, clinical outcomes, adrenal function and brain injury in children undergoing heart surgery with use of CPB.

## Results

### Corticosteroids, inflammation, and clinical outcomes

Many studies have attempted to correlate markers of inflammation after glucocorticoid administration with clinical outcomes [13–26]. Firstly, it is well known that SIRS is a *multifaceted*, complex response that is challenging to characterize and modulate. Therefore, measuring only a few cytokines might not be accurate enough in view of the complex array of pro-inflammatory and anti-inflammatory mediators that are released during the SIRS process [27]. Secondly, while glucocorticoids can blunt inflammation, but this does not necessarily translate into improved short-term clinical outcomes [17, 25, 28]. Moreover, a study by Gessler et al. found no impact of glucocorticoid administration on markers of inflammation [18]. Graham et al. in an RCT of 68 children undergoing surgery with use of CPB found no effect single vs. 2-dose corticosteroids on inflammation [29]. Finally, there is some evidence that the *host inflammatory response* plays an important role and that clinicians should approach SIRS in a personalized rather than standardized manner. Huber et al. [30], in a study of 37 children undergoing heart surgery with use of CPB, demonstrated that the neutrophil phenotype signature could predict end-organ dysfunction associated with SIRS.

Several small-sized RCTs have focused on the effect of corticosteroids on clinical outcomes in the pediatric population (Table 1). Most of these trials measure inflammation parameters and the clinical data is measured either as a primary or secondary outcome. All have in common a small sample size and significant variability in the type and regimen of corticosteroid used. The first randomized controlled trial of corticosteroid (47 children) vs. placebo (48 children) was published by Toledo-Pereyra et al. in 1980. The authors reported improved survival in patients administered methylprednisolone 30 mg/kg: 1 h preoperatively, 5 min pre-CPB and every 6 h for 24 h; however, there was no correlation with other biochemical markers [31]. Bronicki et al. in an RCT of 29 children, found dexamethasone given 1 mg/kg, 1 h prior to CPB, was associated with lower alveolar-arterial gradients, lower fluid requirement and less mechanical ventilation [32]. In an RCT of 30 children, Varan et al. compared the dose-effect of methylprednisolone 30 mg/kg to methylprednisolone 2 mg/kg both given by intravenous infusion pre-CPB. There was no difference in clinical outcomes between the two groups (ventilation time, intensive care unit stay or oxygenation) [15]. Later, Lindberg et al. in an RCT of 40 children found no effect of dexamethasone 1 mg/kg after anesthesia induction on oxygenation, fluid balance, critical care stay, or ventilation time [17]. Schroeder et al in an RCT of 29 children, compared the effect of 30 mg/kg of methylprednisolone double-regimen (pre-operatively and intraoperatively) to a single dose (30 mg/kg) intraoperatively. The double-dose steroid recipients had better oxygenation, lower body temperature and reduced fluid requirements [16]. Checchia et al. reported in an RCT of 28 children no effect dexamethasone 1 mg/kg, given 1-h pre-CPB on sternal dehiscence rates, reoperation for bleeding, or gastrointestinal bleeding [33]. Ando et al. in small RCT of 20 patients found a benefit of hydrocortisone administration in terms of reduced body edema, improved oxygenation and reduced duration of ventilation [34]. Graham et al. randomized a total of 76 patients to either single (intraoperative) or double dose methylprednisolone (8-h pre-operative and intraoperative). The two-dose steroid group had a higher serum creatinine and poorer postoperative diuresis [20]. There was no difference in the incidence of low cardiac output syndrome, inotropic requirement, duration of mechanical ventilation, ICU, or hospital stay. Heying et al. evaluated the effect of dexamethasone 1 mg/kg 4 h pre-CPB in 20 neonates undergoing arterial switch and found no effect of corticosteroids in terms of postoperative cardiac parameters (heart rate, mean arterial pressure, central venous pressure), diuresis, or oxygenation. However, there was reduced dobutamine requirement 4 h post CPB [23]. Keski-Nisula et al. in an RCT of 40 neonates undergoing surgery with use of CPB, found no effect of 30 mg/kg of methylprednisolone given at induction of anesthesia on postoperative lactate, central venous saturation, inotropic score, duration of ventilation, or survival; however, the steroid arm had significantly increased blood glucose [25]. The same group investigated in a three-arm RCT of 45 children the effect of 30 mg/kg methylprednisolone after induction vs. 30 mg/kg methylprednisolone in prime vs. placebo. There were no differences between the three groups, in terms of lactate, inotropic score, duration of ventilation and intensive care stay but the steroid-treated arms had higher blood glucose compared to placebo [35]. Amanullah et al. in RCT of 152 children investigated the effect of dexamethasone 1 mg/kg given at three time-points (induction, pre-CPB and 6 h from the last dose) vs. placebo [28]. There was no difference between the two arms in terms of ventilation times, urine output, mean systolic and diastolic pressure, central venous pressure, inotrope score at 6 h and fluid requirement. An RCT of corticosteroid vs. placebo in 190 neonates undergoing heart surgery with use of CPB, has recently finalized recruitment and hopefully will inform us more on this matter and others [36]. Another larger RCT of 1,200 neonates is currently recruiting patients^1^.

**Table 1:** Randomized controlled trials of steroid use in children.

**First authors**	**Year of publication**	**Sample size**	**Study design**	**Type of steroid**	**Dose**	**Route**	**Regimen**	**Effect steroids on inflammation**	**Effect steroids on clinical outcomes**	**Benefit/no benefit/harm**
Toledo-Pereyra et al. [31]	1980	95 children	RCT steroid vs. placebo	Methylprednisolone	30 mg/kg	IV	Pre-op, pre-CPB, post-CPB, every 6 h for 24 h	Not assessed	Increased survival	Benefit
Bronicki et al. [32]	2000	29 children	RCT steroid vs. placebo	Dexamethasone	1 mg/kg	IV	1-h pre-CPB	Eight-fold decrease in IL-6 levels and a greater than three-fold decrease TNF-α levels after CPB	Less supplemental fluid during the first 48 h, lower alveolar-arterial oxygen gradients during the first 24 h, less mechanical ventilation	Benefit
Varan et al. [15]	2002	30 children	RCT high-dose steroid vs. low-dose steroid	Methylprednisolone	30 vs. 2 mg/kg	IV	30 min IV infusion pre-CPB	No difference in serum IL-6, IL-8, CRP, and polymorphonuclear leukocyte counts	No effect	No benefit
Lindberg et al. [17]	2003	40 children	RCT steroid vs. placebo	Dexamethasone	1 mg/kg	IV	Post anesthesia induction	Decrease in CRP but no change in von Willebrand factor antigen and S100B	No effect	No benefit
Schroeder et al. [16]	2003	29 children	RCT single dose steroid vs. double dose	Methylprednisolone	30 mg/kg	IV	2 dose (4 h before bypass and in bypass prime) vs. 1 dose (intraoperative)	2 dose regimen reduced myocardial mRNA expression for IL-6, MCP-1, and ICAM-1 both before and after bypass and had lower serum IL-6 and increased IL-10 at end-bypass vs. 1 dose	Combined steroid administration reduced fluid requirements, resulted in lower body temperature, and lower arteriovenous oxygen difference	Benefit
Checchia et al. [33]	2003	28 children	RCT steroid vs. placebo	Dexamethasone	1 mg/kg	IV	Pre-CPB	Not assessed	No effect	No benefit
Ando et al. [34]	2005	20 neonates	RCT steroid vs. placebo	Hydrocortisone sodium succinate after discontinuation of CPB	0.18 mg/kg/h for 3 days, 0.09 mg/kg/h for 2 days and 0.045 mg/kg/h for 2 days	IV	Post-CPB	No effect	Less fluid retention, better oxygenation and shorted ventilation times	Benefit
Graham et al. [20]	2011	76 neonates	RCT single dose steroid vs. double dose steroid	Methylprednisolone	30 mg/kg	IV and prime	8 h pre-CPB, IV, and in prime vs. 8 h IV preoperative only	Preoperative interleukin-6 were reduced by two-fold in the 2-dose steroid group	Two-dose methylprednisolone regimen was associated with a higher serum creatinine and poorer postoperative diuresis	Harm
Heying et al. [23]	2012	20 neonates	RCT steroid vs. placebo	Dexamethasone	1 mg/kg	IV	4 h pre-CPB	Decrease in myocardial expression of IL-6, IL-8, IL-1β, and TNF-α mRNA and decrease in protein synthesis of TNF-α; serum IL-6 significantly lower, IL-10 significantly higher in steroid-treated patients; lipopolysaccharide binding protein significantly higher postoperatively treated patient	Steroid recipients had a lower dobutamine requirement 4 h post CPB	Benefit
Keski-Nisula et al. [25]	2013	40 neonates	RCT steroid vs. placebo	Methylprednisolone	30 mg/kg	IV	After induction of anesthesia	Decrease in IL-6 and IL-8 and raised levels of anti-inflammatory IL-10 in steroid-treated patients	Blood glucose higher in the steroid-treated patients	No benefit
Keski-Nisula et al. [35]	2015	45 children	RCT IV steroid vs. in prime steroid vs. placebo	Methylprednisolone	30 mg/kg	IV vs. in prime	After induction vs. in prime	Lower IL-8 levels on weaning and 6 h post-CPB vs. placebo	Blood glucose higher in the steroid-treated patient	No benefit
Amanullah et al. [28]	2016	152 patients (1 month up to 18 years old)	RCT steroid vs. placebo	Dexamethasone	1 mg/kg (maximum dose 12 mg)	IV	At induction of anesthesia—pre-operatively; at the time of initiation of cardiopulmonary bypass—intraoperatively; and 6 h after the second dose—post-operatively	IL-6 were lower at 6 and 24 h postoperatively and IL-10 levels were higher 6 h postoperatively in the steroid group	No effect	No benefit

*Effects on inflammation and/or clinical outcomes. RCT, randomized controlled trial; IV, intravenous; CPB, cardiopulmonary bypass; IL-, interleukin; TNF –α, tumor necrosis factor alpha; CRP, C reactive protein, S100B, calcium-binding protein B, ICAM1, intercellular adhesion molecule 1; MCP1, monocyte chemotactic protein 1*.

Several studies have attempted to quantify the effect of glucocorticoids on markers of myocardial injury [23, 25, 33, 35, 37, 38].However, measuring these markers around the time of cardiac surgery is both difficult to interpret and correlate with clinical outcomes (see Table 2). In the adult population, the SIRS trial demonstrated that the corticosteroid treated arm had an increase in myocardial injury as measured by elevation of the CK-MB enzyme compared with placebo [9].

**Table 2:** Studies of the effect of steroids on markers of myocardial injury.

**First authors**	**Publication Year**	**Sample size**	**Study design**	**Type of steroid**	**Dose mg/kg**	**Route**	**Regimen**	**Cardiac marker**	**Effect on myocardial injury markers**	**Effect on clinical outcomes**	**Cardioprotective**
Checchia et al. [33]	2003	28 children	RCT steroid vs. placebo	Dexamethasone	1 mg/kg	IV	1-h pre-CPB	cTnI	cTnI reduced at 24 h postoperatively in the dexamethasone group	No effect	Yes
Malagon et al. [37]	2005	RCT steroid vs. placebo	RCT steroid vs. placebo	Dexamethasone	1 mg/kg	IV	During anesthesia induction	cTnT	Decrease in cTnT, 8 h after admission	No effect	Yes
Heying et al. [23]	2012	RCT steroid vs. placebo	20 neonates	Dexamethasone	1 mg/kg	1 mg/kg	4-h pre-CPB	cTnT	cTnT lower in dexamethasone-treated patients, 1 h postoperatively	Lower dobutamine requirement	Yes
Keski-Nisula et al. [25]	2013	40 neonates	RCT steroid vs. placebo	Methylprednisolone	30 mg/kg	IV	After anesthesia induction	cTnT	No effect	No effect	No
Keski-Nisula et al. [35]	2015	45 children	RCT IV steroid vs. in prime steroid vs. placebo	Methylprednisolone	30 mg/kg	IV vs. prime	After anesthesia induction vs. in prime	cTnT	cTnT lower in steroid recipients, both at induction and in prime steroid vs. placebo, 6 h post CPB wean	Higher blood glucose in steroid-treated groups	No
Pesonen et al. [38]	2017	45 children	RCT of steroid IV vs. in the prime vs. placebo	Methylprednisolone	30 mg/kg	IV vs. in prime	After induction vs. in prime	Plasma heart-type fatty-acid-binding protein and cTnT	Plasma heart-type fatty-acid-binding protein and TnT decreased 6 h post-op in both steroid regimens	No effect	Yes

*RCT, randomized controlled trial; CPB, cardiopulmonary by-pass; IV, intravenous; cTnI, cardiac troponin I; cTnT, cardiac troponin T*.

In view of the limitations of the existing RCT evidence discussed above, the available meta-analyses should be interpreted with caution. The Cochrane meta-analysis by Robertson-Malt et al. concluded that corticosteroids do not significantly reduce post-operative complications as measured by length of stay in ICU, peak core temperature and duration of ventilation [39]. A more recent meta-analysis by Scrascia et al. concluded no significant effect of corticosteroids on mortality, mechanical ventilation time or ICU length of stay. Based only on 15 patients, the authors found a reduced prevalence of renal dysfunction associated with the use of steroid (13 vs. 2 patients) [40].

In addition to the number of small-sized randomized trials already discussed, several larger observational studies are worth discussing here (Table 3). The Pasquali et al. [41, 42] studies provide us with the largest sample despite their retrospective design. In a registry data-base study of 46,730 patients, of which 54% received perioperative corticosteroids, there was no difference in mortality or ventilation times, however steroid use was associated with increased length of stay, higher incidence of infection and greater use of insulin. In the analysis stratified by a congenital cardiac risk score (the RACHS-1 score), increased morbidity associated with steroid use was more evident in the lower risk categories (e.g., 1–3) [41]. Later, the same group published a multicenter database study focused on 3,180 neonates and found corticosteroids to be associated with increased infection across all regimens in the lower risk groups. [42]. An observational study by Mastropietro et al. on 76 children undergoing complex heart surgery found that a greater cumulative duration of steroid administration was associated with higher risk of postoperative infection [43].

**Table 3:** Retrospective studies of the effect of steroids on clinical outcomes.

**First authors**	**Year of publication**	**Sample size**	**Study design**	**Type of steroid**	**Dose**	**Route**	**Regimen/timing**	**Effect steroids on clinical outcomes**	**Benefit/no benefit/harm**
Pasquali et al. [41]	2010	46,730 children	Retrospective	Variable (methylprednisolone, prednisolone, dexamethasone, or hydrocortisone)	No data	No data	On the day before or day of surgery	Corticosteroids were associated with longer length of stay, greater infection, greater use of insulin, increased morbidity was most prominent in RACHS-1 categories 1 through 3 (lower risk groups)	Harm
Pasquali et al. [42]	2011	3,180 neonates	Retrospective	Methylprednisolone	No data	No data	Methylprednisolone on the day before surgery and the day of surgery, day of surgery only and the day before surgery only	In lower surgical risk groups, there was a significant association of methylprednisolone with infection across all regimens	Harm
Mastropietro et al. [43]	2013	76 children	Retrospective	Methylprednisolone, hydrocortisone, dexamethasone	All patients had methylprednisolone 30 mg/kg, hydrocortisone 1 mg/kg (48% patients), dexamethasone 0.5 mg/kg (86% patients)	IV	Methylprednisolone before surgical incision, hydrocortisone 6 h for hemodynamic instability, periextubation dexamethasone every 6 h	Greater cumulative duration of corticosteroid exposure was independently associated with postoperative infection	Harm
Dreher et al. [46]	2015	525 children	Retrospective (non-steroid cohort vs. steroid cohort, 6 months prior steroid discontinuation)	Methylprednisolone	30 mg/kg up to a maximum dose of 500 mg	In the prime	In the prime	Steroids group had more postoperative wound infection and respiratory failure requiring tracheostomy	Harm
Elhoff et al. [47]	2016	549 neonates	Retrospective	No data	No data	No data	Intraoperative	Improved hospital survival in the non-steroid group	Harm

*IV, intravenous; RACHS-1, risk adjustment in congenital heart surgery*.

Indeed, neonates have been the focus of multiple studies because they are considered to be the most vulnerable to CPB insult. This may be due not only to the immaturity of the HPA axis [11] but neonates also appear to display a distinct inflammatory response compared to older age groups [44]. However, a recent best evidence topic review by our group could not find a clear clinical benefit of corticosteroid in neonates mainly due to the limitations in the available evidence, as discussed earlier [45].

Dreher et al. [46] in a retrospective single-center database study, compared the effect of methylprednisolone administration in 303 children undergoing heart surgery during the 6 months prior to discontinuation of steroid use with a cohort of 222 children were no glucocorticoid was used. Overall, the steroid group had more wound infection and more respiratory failure requiring tracheostomy. There were no differences in the rest of the clinical outcomes (early mortality, ventilation time, renal failure etc.,). In the neonate subgroup analyses, no difference in clinical outcomes was detected. Using datasets from a clinical trial (the Pediatric Heart Network's Single Ventricle Reconstruction trial), on neonates that had the Norwood procedure, Elhoff et al. [47] compared the effect of intraoperative corticosteroid administration in 498 recipients with 51 non-recipients and found in the multivariate analysis no effect of corticosteroid on lengths of stay but a trend toward better hospital survival in non-recipients. This is one of the few studies where the authors have also looked at the neurodevelopment outcomes at 14 months and found no difference. A major limitation in the current studies of the impact of corticosteroids on the clinical outcomes of pediatric heart surgery is the lack of long-term follow-up of the effect of corticosteroid administration on late neurocognitive outcomes. In other patient groups, the early administration of corticosteroids had detrimental effects. In an RCT of 262 infants with severe respiratory distress syndrome requiring mechanical ventilation, dexamethasone 0.25 mg/kg given every 12 h for 1 week was associated with adverse effects on the neuromotor and cognitive function at school age [48].

In conclusion, most studies conclude that corticosteroids dampen the SIRS to surgery. The effect of corticosteroids on clinical outcomes has been studied in several small-sized RCTs with conflicting evidence. Some studies show a beneficial effect on clinical outcomes such as ventilation, oxygenation or renal function parameters while in other studies, the use of corticosteroids had no effect at all. Apart from being small-sample sized, these studies used various types of steroid and regimens, therefore, it is difficult to draw any valid conclusions. A few large registry studies have raised concerns about the association of corticosteroid administration with infection, however, their results remain limited by their retrospective design.

### Relative adrenal insufficiency

Another justification for the use of corticosteroids is the so-called *relative adrenal insufficiency* or *adrenal cortex exhaustion*, originally described during critical illness or sepsis. According to this definition, the adrenal response and subsequent concentration of plasma cortisol is not adequate to cope with the stress of surgery [49, 50]. Therefore, it is thought that perioperative steroid supplementation could compensate for this potential deficit. However, defining relative adrenal insufficiency during critical illness remains a matter of debate. The most commonly used diagnostic criteria in the literature for adrenal insufficiency during critical illness or surgery is the ACTH stimulation test described by Anane et al. In this test, synthetic ACTH (Synacthen, cosyntropin, tetracosactrin) is given intravenously. Cortisol is measured before Synacthen injection and after, at 30 and 60 min respectively. In 189 consecutive adult patients with septic shock, the authors found that an incremental cortisol response to ACTH (defined as the difference between the basal cortisol and the highest value between cortisol measured at 30 and 60 min) of <9 μg/dL or a high baseline cortisol concentration (>34 μg/dL) were of good prognostic value for identifying patients at risk of death [50]. However, this test is based on a few time-point value measurements of cortisol. Therefore, this could prove inaccurate in the context of a dynamic, pulsatile cortisol secretion that was described in both healthy volunteers or adults undergoing heart surgery [51, 52]. The major limitation of the current literature aimed at understanding the HPA axis function during pediatric heart surgery is to try to correlate the findings of the ACTH test with the measures of clinical outcome. Other limitations include the measurement of only a few, random plasma cortisol levels, the variability in the dose used for Synacthen testing and most importantly the concomitant use of glucocorticoids at induction that obscures the assessment of the HPA axis [11, 12, 53–65]. Adequate assessment of the HPA axis during surgery and critical illness requires very frequent cortisol measurements [66].

Several studies have attempted to characterize dynamic HPA axis physiology during surgery or critical illness in children (Table 4). Kucera et al measured plasma cortisol at six time points in 24 children of various ages (ranging from 2 months to 15 years): the day before surgery, at the end of surface cooling, at the lowest temperature during CPB, 10 min after rewarming, at the end of CPB and on the 8th day, postoperatively [67]. They found the cortisol levels to be in the range of the normal laboratory values with a trend toward increase during rewarming. Anand et al measured cortisol levels at seven time points in 15 neonates: pre-operatively, pre-CPB, during DHCA, at 6, 12, and at 24 h. The peaks in cortisol secretion were recorded before CPB and at the end of the operation, then, the cortisol levels fell below preoperative values at 12 h [68]. Gajarski et al. measured cortisol at 10 time-points in 58 children: baseline—preoperative, time of surgery and every 6 h up to 48 h. They have stratified the cortisol profiles by various patient groups: control (non-bypass or non-cardiac surgery procedures), CPB only (no DHCA), DHCA (no steroid used), and DHCA with steroid administration. The cortisol and ACTH peaked within 2 h of surgery but without differences between the groups. In 9 patients, not from the DHCA-steroid group, they noted an elevated ACTH-cortisol ratio that correlated with an elevated inotropic score postoperatively [53]. In 21 neonates undergoing heart surgery with use of CPB, Garcia et al. assessed the HPA axis with a low dose ACTH stimulation test (1 μg) on day 1 postoperatively. The patients that had a significant serum cortisol increase after ACTH test (>50 mg/dL) also had a higher mean arterial blood pressure at 48 h postoperatively. All patients included in the study had dexamethasone 0.5 mg/kg midnight before surgery and at induction [54]. In 38 neonates undergoing complex heart surgery, Mackie et al. measured serum cortisol at 3 time-points: preoperatively, at 24 and 48 h postoperatively. They found the higher cortisol levels to be associated with greater atrial filling pressure and lower cardiac index. Again, all patients received methylprednisolone (30 mg/kg) at induction [55]. Wald et al. [56] measured Synacthen stimulated total cortisol and corticosteroid binding globulin levels preoperatively and postoperatively in 51 children undergoing surgery with use of CPB (subjects aged <2 years received 125 μg while subjects >2 years received 250 μg). Lower CBG and increased free cortisol (>6 μg/ml) correlated with worse clinical outcomes (longer length of stay, longer ventilation time, increased fluid requirement). The authors found that only 17.6% of the patient had a low baseline total cortisol and all had a “normal” response to Synacthen. They concluded that total cortisol is not a good measure of HPA axis function in this setting. All patient received 1 mg/kg of dexamethasone before CPB. Verweij et al. [64] in a retrospective analysis of 62 patients with low cardiac output post pediatric heart surgery, found no effect of hydrocortisone administration in patients with supposed adrenal insufficiency (defined as basal cortisol <100 nmol/l). Sasser et al. [65] in a retrospective analysis of 41 neonates found that a postoperative serum cortisol level >10 μg/dL was not associated with worse clinical outcomes (lactate measurements, inotropic score, fluid requirement, arteriovenous saturation difference, mean blood pressure, mean CV, mean heart rate or ventilation time). Furthermore, there was no difference in steroid responsiveness compared to the cortisol >10 μg/dL group, among patients that had hemodynamic compromise and were administered hydrocortisone, postoperatively. In a prospective analysis of 119 children undergoing heart surgery Schiller et al. measured cortisol levels pre-operatively and at 18 h after surgery. All patients were given methylprednisolone (30 mg/kg) at induction [63]. The authors defined adrenal insufficiency as a measured postoperative cortisol level of <18.1 μg/dL or a delta cortisol value of <9 μg/dL (e.g., postoperative cortisol - preoperative cortisol). There was no significant correlation between patients with adrenal insufficiency by this definition and the procedure complexity (low or high). Furthermore, the postoperative course (ICU stay ventilation time, lactate, urine output) of children with adrenal insufficiency was not different from the children with apparently normal adrenal function. A study by Bangalore et al. assessed postoperative serum cortisol levels at three time-points: immediately after surgery and in the first and second postoperative mornings. The cortisol fell significantly over the first 24 h. Higher postoperative cortisol measurements were associated with increased morbidity. All patients received methylprednisolone (30 mg/kg) at induction [62]. Crow et al measured serum cortisol and dexamethasone following 1 mg/kg of dexamethasone in 32 infants undergoing cardiac surgery with CPB. They noted significant variability in dexamethasone levels and grouped the patients into high dexamethasone (>15 mg/dL) or low dexamethasone (<15 mg/dL) on arrival to ICU. Although the patients that had a higher dexamethasone levels had more pronounced suppression of endogenous cortisol compared to basal levels this did not translate into any impact on clinical outcomes [61]. Teagarden et al. reviewed 24 patients that underwent surgery for congenital heart disease and found that lower pre-hydrocortisone cortisol concentrations were associated with improved hemodynamics after hydrocortisone administration [58]. Maeda et al. classified 32 neonates undergoing heart surgery into patients with adrenal insufficiency (baseline cortisol <15 μg/dL or incremental increase after testing of <9 μg/dL and baseline cortisol of 15–34 μg/dL) and a group with normal adrenal function, after ACTH test (3.5 μg/kg of tetracosactide acetate). All patients received perioperatively 1 mg/kg hydrocortisone every 6 h up to 18 h from first hydrocortisone dose. Only the patients diagnosed with adrenal insufficiency exhibited a significant increase in mean blood pressure and urine output in response to hydrocortisone administration [59]. A recent study by Crawford et al. [57] aimed to correlate relative adrenal insufficiency to clinical outcomes in 40 neonates undergoing complex heart surgery. Like the studies discussed above, all patients received preoperative methylprednisolone. The authors defined adrenal insufficiency as <9 μg/ml increase in cortisol at 30 min post ACTH test (cosyntropin 1 μg). Five percent of the patients had adrenal insufficiency post-CPB and this was significantly associated with increased serum lactate and higher inotrope requirement.

**Table 4:** Studies aimed at understanding the hypothalamic pituitary adrenal axis function in children.

**First authors**	**Year**	**Sample size**	**Study design**	**Steroids perioperatively**	**Cortisol time-point number**	**Frequency of cortisol measurement**	**ACTH test/measurement**	**ACTH test dose**	**Definition of adrenal insufficiency used in the study**	**Correlation to clinical outcome**	**Take home message of the study**
Kucera et al. [67]	1986	24 children	Observational	No data	6	1 time-point day before surgery, 4 time-points on the day of surgery, and 1 timepoint 8th day of surgery	No	NA	NA	Not assessed	Cortisol levels in the range of the normal laboratory values with a slight increase during rewarming
Anand et al. [68]	1990	15 neonates	Observational	No	7	Pre-op, pre-CPB, during DHCA, end of the operation, 6, 12, and 24 h	No	NA	NA	Survival rate	Non-survivors (*n* = 4) had higher concentrations of cortisol than survivors
Gajarski et al. [53]	2010	58 children	Observational	10 patients in the DHCA arrest group	10	Before surgery, after surgery, 6, 12, 18, 24, 30, 36, 42, and 48 h	ACTH measured at same time points with cortisol	NA	ACTH/serum cortisol ratio cut off >15	Peak cortisol level did not correlate with simultaneous inotrope score; nine patients had increased ACTH/cortisol ratios in association with elevated inotrope requirement (none of these patients had steroids)	Cortisol peaked within 2 h of surgery; ACTH inversely correlated with bypass time in patients with DHCA but not with circulatory arrest time
Garcia et al. [54]	2010	21 neonates	Retrospective	All patients received dexamethasone 0.5 mg/kg the night before surgery	2	Basal and post ACTH test	ACTH test, first post-op day in patients with worsening hemodynamic status	1 μg	Basal cortisol post ACTH <30 μg/dL post ACTH test	All neonates with hemodynamic instability had low basal serum cortisol; 48 h post-surgery the mean arterial pressure in the groups with cortisol >50 μg/dL post ACTH stimulation was significantly higher than the patient with cortisol <50 μg/dL	Cortisol level cut off of ≤20 mg/dL may not be applicable in neonates undergoing heart surgery to diagnose AI
Mackie et al. [55]	2011	38 neonates	Observational	All patients had methylprednisolone, 30 mg/kg, IV at anesthetic induction	3	Preoperative, at 24 and 48 h post-surgery	No	NA	None	Higher cortisol levels were associated with greater atrial filling pressures and a lower cardiac index	Relation between serum cortisol and cardiovascular system warrants further research
Wald et al [56]	2011	52 children	Observational	All patient had received 1 mg/kg dexamethasone before CPB, not to exceed a 10 mg total dose	2	Pre - and postoperative	No	Cosyntropin: 250 μg for children >2 years of age and 125 μg for children <2 years	Reference range for normal total plasma cortisol was 3–21 μg/dL. Basal free cortisol in critical illness was defined as >2.0 g/dL. A normal free cortisol value after cosyntropin test was defined as >3.1 μg/dL and total serum cortisol increase ≥9 μg/dL	9 patient had low total cortisol (<3 μg/dL) baseline but normal ACTH stimulation test). Patient with free cortisol increase difference of >6 μg/dL had a longer length of stay, higher inotrope scores, greater fluid requirement, longer ventilator times	Using total cortisol to investigate AI may be inadequate. Decreased corticosteroid binding globulin levels post ACTH stimulation associated with worse clinical outcomes
Verweij et al. [64]	2012	62 children with low cardiac output	Retrospective	All patients had dexamethasone 0.5 mg/kg before surgery	1	Basal cortisol	No	NA	Baseline value of total cortisol of <100 nmol/l	Similar effect of hydrocortisone in in the groups with low or normal basal cortisol levels	Baseline value of total cortisol of <100 nmol/l not adequate to define adrenal insufficiency
Sasser et al. [65]	2012	41 neonates	Retrospective	All the patients received 10 mg/kg methylprednisolone 8 and 1 h before transport to the operating room and 15 with hemodynamic compromise received 100 mg/m2/d hydrocortisone	1	Postoperative (on arrival to intensive care unit)	No	NA	Postoperative cortisol <10 mg/dL	Postoperative cortisol <10 mg/dL not associated with worse clinical outcomes, 6 out of 15 patients responded to steroid but there was no difference in the levels of cortisol between responders and non-responders.	Use of absolute cortisol threshold is not useful in identifying AI
Schiller et al. [63]	2013	119 children	Observational	All patients received intravenous methylprednisolone, 30 mg/kg to a maximum dose of 300 mg/kg, at induction	2	Before and 18 h after surgery	No	NA	Postoperative cortisol level <18.1 μg/dL or delta cortisol (postoperative cortisol - preoperative cortisol) <9 μg/dL	Normal adrenal function subgroup had greater inotropic support at 12, 24, and 36 h after surgery and a higher lactate level at 12 and 24 h after surgery; no differences in outcomes between patients with AI and normal adrenal function in the first 36 h, no correlation between AI and procedure complexity	AI does not translate into worse clinical outcomes
Bangalore et al. [62]	2014	33 neonates	Observational	All patients methylprednisolone, 20 mg/kg, at induction	3	Day 0 (after intensive care until admission); day 1 (first morning of surgery), day 2 (second morning of surgery)	No	NA	NA	Higher cortisol was associated with greater morbidity, including the need for preoperative ventilation, increased total duration of ventilation, duration of inotropic support, and hospital length of stay	High postoperative cortisol was associated with increased post-operative morbidity
Crow at al. [61]	2014	32 infants	Observational	1 mg/kg of dexamethasone before cardiopulmonary bypass (CPB) initiation.	7	After anesthesia induction, after CPB, after intensive care unit (ICU) arrival, and 4, 8, 12, and 24 h after surgery; dexamethasone levels on intensive care unit arrival	ACTH measured at same time points with cortisol	NA	NA	No difference in clinical outcomes between patient with high dexamethasone levels (≥15 mg/dL) and low dexamethasone levels (≤15 mg/dL); cortisol levels remained low throughout the first 24 postoperative hours even after dexamethasone levels neared zero.	Dexamethasone levels are highly variable despite a standardized administration protocol
Teagarden et al. [58]	2016	24 patients (<21 years), median age = 1.4 months (range 0.1–232 months)	Retrospective	Intra- operative dose of methylprednisolone (30 mg/kg) before surgical incision and 1 mg/kg intravenously every 6 h for patients with hemodynamic instability	1	Pre-hydrocortisone treatment serum cortisol	No	NA	Favorable responders were defined as patients in whom, at 24 h after hydrocortisone initiation, either (1) systolic blood pressure was increased or unchanged and vasoactive- inotrope score was decreased or (2) systolic blood pressure increased by ≥10% of baseline and vasoactive-inotrope score was unchanged	Serum cortisol obtained before initiation of hydrocortisone was significantly lower in patients who responded favorably to the postoperative hydrocortisone	Total serum cortisol helpful in identifying children recovering from cardiac surgery who may or may not hemodynamically improve with hydrocortisone
Maeda et al. [59]	2016	32 neonates	Retrospective	Hydrocortisone 1 mg/kg, was given every 6 h immediately after ACTH test	3	Baseline and at 30 and 60 min after the tetracosactide stimulation	Yes	3.5 μg/kg of tetracosactide acetate	baseline cortisol <15 μg/dL or incremental increase after testing of <9 μg/dL	One-fifth of infants developed adrenal insufficiency, steroid administration in these patients resulted in a significant increase in blood pressure and urine output	Steroid replacement therapy improved hemodynamics only in the subgroup with adrenal insufficiency
Crawford et al. [57]	2016	40 neonates	Retrospective	Methylprednisolone 10 mg/kg 8 h and 1 h	2	Basal and 30 min post ACTH test	ACTH measured at same time points with cortisol	1 μg cosyntropin the day prior to surgery before preoperative methyl- prednisolone; and the second, 1 h after separation from CPB	AI was defined as <9 μg/dL increase in cortisol at 30 min post ACTH test	32.5% had AI post CPB, AI was associated with increased median colloid resuscitation, higher serum lactate	AI determined by a low dose ACTH test occurs in one third of patient and is not affected by pre-operative steroid administration

*CPB, cardiopulmonary by-pass; DHCA, deep hypothermic circulatory arrest; ACTH, adrenocorticotrophic hormone; NA, not applicable; AI, adrenal insufficiency; NA, not applicable*.

We still do not have an accepted definition of adrenal insufficiency in children undergoing heart surgery, hence it is difficult to draw conclusions about the effect of corticosteroids on adrenal function and clinical outcomes. Most of the studies so far have used very few time-point measurements, with conflicting results and were undertaken in children that received corticosteroids at induction, therefore, making an accurate assessment of the HPA axis almost impossible.

### Corticosteroids and cerebral protection

Cardiac surgery with use of deep hypothermic circulatory arrest is known to be associated with impaired cerebral oxygen metabolism and cerebral edema [69, 70]. Corticosteroids are used to treat cerebral edema secondary to brain tumors [71] but are also used in the context of head trauma [72]. Therefore, another justification for the use of corticosteroids is the use for DHCA surgery. However, the CRASH trial showed an increase in death at 2 weeks and 6 months with the use of corticosteroids [72, 73]. The evidence of the impact of corticosteroids on brain protection during the use of DHCA has not been studied extensively and is limited to *in vitro* studies [74] and a few *in vivo* experiments on piglets [69, 75] with conflicting results.

Schmitt et al. investigated the effect of deep hypothermic circulatory arrest on an *in vitro* model of mouse neonatal astrocytes, neurons, and BV-2 microglia cells. The effect of methylprednisolone (100 nM) was tested in cells that were incubated according to a protocol that mimics the temperature changes during pediatric deep hypothermic circulatory arrest: deep hypothermia, slow rewarming and normothermia. The authors measured in all cell lines the cytotoxicity and the production of IL-6 as a marker for neuroprotection and regeneration. While steroid administration had no effect in the normothermic treated cells, in the deep hypothermia-treated cells methylprednisolone increased cell survival but decreased the protective IL-6 [74].

Langley et al. randomized two groups of 8 piglets to placebo and intramuscular methylprednisolone 30 mg/kg, given 8- and 2 h before induction. All piglets underwent cooling to 18°C, 60 min of circulatory arrest and 60 min of reperfusion and rewarming. The steroid-treated arm had a significantly higher recovery of cerebral blood flow and cerebral oxygen metabolism [69]. In a later study, Schubert et al. randomized two groups of seven neonatal piglets to intravenous methylprednisolone (30 mg/kg), given 24 h before surgery vs. placebo. The piglets were then cooled to 15°C for a longer period of DHCA-−120 min–before rewarming for 40 min. The authors conducted quantitative histological studies in the hippocampus, cortex, cerebellum and caudate nucleus. Piglets in the steroid-treated arm had increased neuronal cell death and apoptosis in the dentate gyrus of the hippocampus.

The neuroprotective potential of corticosteroids in cases of pediatric heart surgery with use of deep hypothermic circulatory arrest has therefore been the least studied. Current evidence is contradictory and the results from the available animal studies highlight the need for robust human trials aimed at this high-risk patient subgroup.

## Conclusions

We found no clear evidence that the anti-inflammatory effect of corticosteroids translates into better clinical outcomes. Most randomized studies in the literature report too few patients, different endpoints and vary widely in the steroid type, doses, and regimens. Although some registry studies examined the effect of corticosteroids on larger patient populations, they are limited by their retrospective design. The effect of corticosteroids on clinical outcomes will need to be clarified by a large, multicenter, randomized controlled trial with clear agreed methodology and aims. Our knowledge about the basic physiology of the hypothalamic-pituitary-adrenal axis during surgery remains limited and it is unclear how to define relative or absolute adrenal insufficiency in the context of pediatric heart surgery. To comprehend this will require understanding of the dynamic, pulsatile secretion of adrenal cortisol which can only be gained from studies that use frequent time-point measurements in patients not receiving exogenous corticosteroids. Finally, the neuroprotective effect of corticosteroids during deep hypothermic circulatory arrest remains even more controversial and warrants further research.

## Author contributions

DF: literature review, writing, design, supervision; BG, TU, SS, MC, and SL: writing of manuscript sections, revision; GA: writing, design, supervision.

### Conflict of interest statement

The authors declare that the research was conducted in the absence of any commercial or financial relationships that could be construed as a potential conflict of interest. The reviewer EA and handling Editor declared their shared affiliation.

## Footnotes

^1^STeroids to REduce Systemic Inflammation After Neonatal Heart Surgery (STRESS). Available online at: https://clinicaltrials.gov/ct2/show/NCT03229538
